# The Art of PEGylation: From Simple Polymer to Sophisticated Drug Delivery System

**DOI:** 10.3390/ijms26073102

**Published:** 2025-03-27

**Authors:** Davit Makharadze, Luis J. del Valle, Ramaz Katsarava, Jordi Puiggalí

**Affiliations:** 1 Departament de Enginyeria Química, Escola d’Enginyeria de Barcelona Est, Universitat Politècnica de Catalunya, Av. Eduard Maristany 10-14, 08019 Barcelona, Spain; davit.makharadze@upc.edu (D.M.); luis.javier.del.valle@upc.edu (L.J.d.V.); 2 Barcelona Research Center in Multiscale Science and Engineering, Universitat Politècnica de Catalunya, Campus Diagonal-Besòs, Av. Eduard Maristany 10-14, 08019 Barcelona, Spain; 3 Institute of Chemistry and Molecular Engineering, Agricultural University of Georgia, Tbilisi 0159, Georgia; r.katsarava@agruni.edu.ge

**Keywords:** PEG, NPs, protein PEGylation, cancer, PEG density

## Abstract

The development of effective drug delivery systems (DDSs) is important for cancer and infectious disease treatment to overcome low bioavailability, rapid clearance and the toxicity of the therapeutic towards non-targeted healthy tissues. This review discusses how PEGylation, the attachment of poly(ethylene glycol) (PEG) molecules to nanoparticles (NPs), enhances drug pharmacokinetics by creating a “stealth effect”. We provide the synthesis methods for several PEG derivatives, their conjugation with NPs, proteins and characterization using modern analytical tools. This paper focuses particularly on covalent conjugation and self-assembly strategies for successful PEGylation and discusses the influence of PEG chain length, density and conformation on drug delivery efficiency. Despite the PEGylation benefits, there are several challenges associated with it, including immunogenicity and reduced therapeutic efficacy due to accelerated blood clearance. Therefore, the balance between PEGylation benefits and its immunogenic risks remains a critical area of investigation.

## 1. Introduction

Cancer remains one of the biggest challenges in medicine to this day, the cure of which is associated with great difficulties despite the current progress in chemotherapy, radiation and targeted therapy. The major obstacle to cancer treatment is the effective delivery of therapeutic drugs while avoiding damage to healthy cells. The low bioavailability and rapid clearance of the medications limit their ability to demonstrate full therapeutic effects. Hence, nanoparticles (NPs) have attracted special interest due to their small size, biocompatibility, biodegradability and ability to encapsulate both hydrophobic and hydrophilic drugs. This versatility of NPs improves drug stability, provides controlled release, and increases circulation time. However, when NPs are in a physiological environment, they interact with the surrounding biological components, such as proteins, lipids and other biomolecules, negatively affecting their functionality [1]. To address NP limitations, PEGylation (i.e., the conjugation with polyethylene glycol (PEG) molecules) has been proposed, enhancing NP stability [2] and therefore action duration, while preventing immune system detection by a phenomenon generally accepted as the “stealth effect”. Anticancer reagents, especially those of a hydrophobic nature, including docetaxel (DTX) and paclitaxel (TXL), are well-known for their low bioavailability, rapid metabolism and high systemic toxicity. NP [3] or direct drug PEGylation [4] has been demonstrated to significantly improve their safety and overall performance. The technology of PEGylation has evolved over several decades; the earliest mentions of PEG can be traced back to the 1940s, when it was referred to as Carbowax. This commercial product was developed by the chemical company Union Carbide (now part of Dow Chemical) and has been used in a wide variety of applications. However, the conjugation of PEG to proteins (i.e., PEGylation) was first performed by Professor Frank Davis and his group at Rutgers University in the late 1970s, aiming to reduce the immunogenicity of the enzyme bovine liver catalase while increasing its circulation half-life. In the following years, his team designed the PEGylated enzyme, bovine adenosine deaminase (ADA), becoming the first FDA-approved PEGylated product (Adagen) for enzyme replacement therapy. Since then, the treatment of many diseases has been improved with PEGylated drugs. In endocrine and metabolic diseases, PEGylation is beneficial for parathyroidism and growth hormone (GH) deficiency. Recently, the FDA authorized Yorvipath, which is a modified palopegteriparatide attached to a branched PEG that enhances the action duration of the parathyroid hormone responsible for calcium homeostasis. Similarly, Skytrofa (lonapegsomatropin) is administered to children with GH deficiency. In the realm of hematologic disorders, PEGylated drugs are employed to treat conditions such as neutropenia and hemophilia. In neutropenia, typically observed after receiving chemotherapy, several PEGylated forms of granulocyte colony-stimulating factor (G-CSF) are used, including Neulasta (pegfilgrastim), Stimufend (pegfilgrastim-fpgk), Fylnetra (pegfilgrastim-pbbk), and Ziextenzo (pegfilgrastim-bmez) improving the production of white blood cells. PEG medications such as Rebinyn (recombinant coagulation factor IX) and Jivi (damoctocog alfa pegol) increase the stability of clotting factors and reduce the required infusion frequency for hemophilia treatment. Finally, a PEGylated aptamer called Macugen (pegaptanib) is often used in ophthalmology to treat macular degeneration, which results in vision loss in older people.

Recent reviews have discussed important elements of PEGylation. The clinical significance of PEGylation technology as well as its benefits are discussed by Gao et al. [5]. Belen et al. provided an analysis of protein PEGylation techniques, emphasizing site-specific PEG conjugation [6]. Similarly, Li et al. reported recent progress in the development of PEGylated therapeutic proteins and peptides [7]. Additionally, current works have debated the challenges of PEGylation, such as the immunogenicity of PEGylated products [8,9]. Although PEGylation has many advantages, there are also some considerable drawbacks, such as the immunogenic response to PEG, commonly known as “anti-PEG antibodies” (APAs), which can accelerate the clearance of PEGylated NPs from the bloodstream. In this review, we provide a comprehensive analysis of PEGylation for drug delivery systems (DDSs) starting from synthetic strategies of various PEG derivatives, their applications in NP and protein PEGylation, pharmacokinetic implications and the safety concerns about PEGylation. In general, a combination of NP-based systems with PEG results in better drug delivery profiles and the goal of this review is to demonstrate the benefits which are associated with PEGylation, particularly for cancer treatment.

## 2. PEG Functionalization

PEG is a hydrophilic, biocompatible polymer widely used in biomedical and pharmaceutical applications because of its unique physicochemical properties, such as high solubility in water and organic solvents, non- or low toxicity, low immunogenicity, cryoprotective capability [10], etc. The PEG terminal hydroxyl group OH-(CH_2_CH_2_O)_n_-H can be modified with various ligands (Table 1). Unmodified PEG is biologically inert, meaning it lacks functional groups that enable specific interactions with biological targets, whereas activated PEG can be covalently attached to drugs [11], proteins [12], or different biomolecules for constructing hydrogels, NPs and other materials that benefit from PEG’s antifouling and stabilizing properties. Functional PEG can be categorized into homobifunctional and heterobifunctional types. Homofunctional PEG refers to both monofunctional (one functional group at the end) or homobifunctional PEG derivatives. The key characteristic of the homobifunctional PEG is identical reactive groups at both ends of the polymer chain, which enables crosslinking, essential in hydrogel preparation [13,14]. Heterobifunctional PEG, on the other hand, is suitable for site-specific conjugation to various substrates or bioactive compounds since there are unique active groups on each terminus.

### 2.1. Synthesis and Functionalization of Amine and Thiol-Terminated PEG

**Figure 1:**
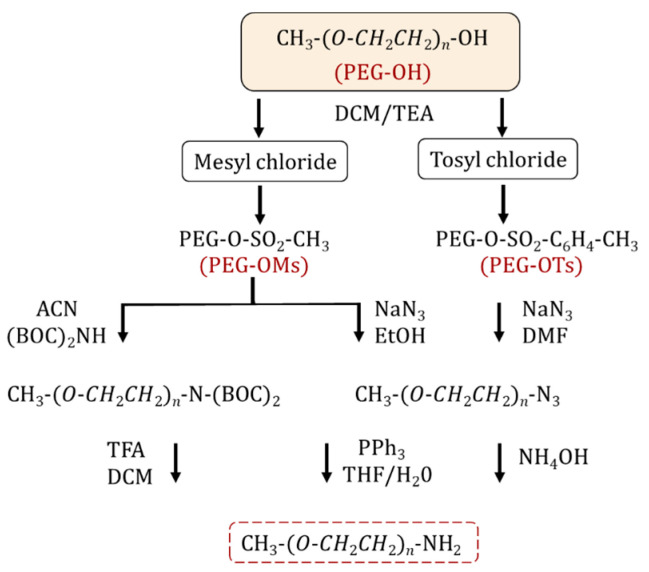
Synthetic pathways for NH_2_-PEG preparation.

The synthesis of an amino-terminated PEG is performed via a two-step approach. Initially, the PEG is activated by halogenation [11,15] or more commonly, sulfonylation (i.e., tosylation, mesylation) [15,16,17,18] through reacting with tosyl (Ts) and mesyl (Ms) chlorides. The resulting PEG intermediates contain effective leaving groups for nucleophilic substitution (Figure 1). The reaction is performed in the presence of organic bases such as pyridine [15] and triethylamine [15,19]. In the second step, the sulfonylated PEG is treated with ammonia to form NH_2_-PEG [20,21,22,23]. However, by-products such as secondary/tertiary amines can be produced as side reactions, which reduce the yield and complicate the purification of the final product. Alternatively, sulfonylated PEG can react with sodium azide to form PEG-N_3_, for the subsequent synthesis of amine-PEG using the Staudinger reaction [20,24,25]. The latter method leads to a PEG product with higher purity and yield compared to the amination process. Zhang et al. reported the synthesis of homofunctional and bifunctional amino-PEG using a 3-step methodology: (1) mesylation; (2) the substitution reaction of activated mPEG-OMs with (Boc)_2_NH to obtain mPEG-N(Boc)_2_ followed by deprotection of the Boc groups with trifluoroacetic acid to obtain mPEG-NH_2_; and (3) finally, the reaction yielded 95–99% purity [26]. Thiol-functionalized PEG is practical for creating self-assembling structures on gold surfaces or other metal surfaces and for protein PEGylation through a Michael-addition reaction. The simplest method to introduce a thiol group into a PEG is a nucleophilic substitution between tosyl-PEG and a thiol nucleophile [27] or an esterification reaction between mercapto acids/mercaptoacetic acid [28], mercaptohexadecanoic acid [29], 3-mercaptopropionic acid and PEG in the presence of sulfuric acid [30,31,32]. Mulay et al. described an environmentally friendly approach for thiol-PEG synthesis using an enzymatic process by transesterification reaction with methyl 3-mercapto propionate in the presence of Candida antarctica lipase B; the reaction yields were approximately 100% for both PEG_1000_ and PEG_2050_ monothiols [33].

### 2.2. Alkyne-Functionalized PEG

**Figure 2:**
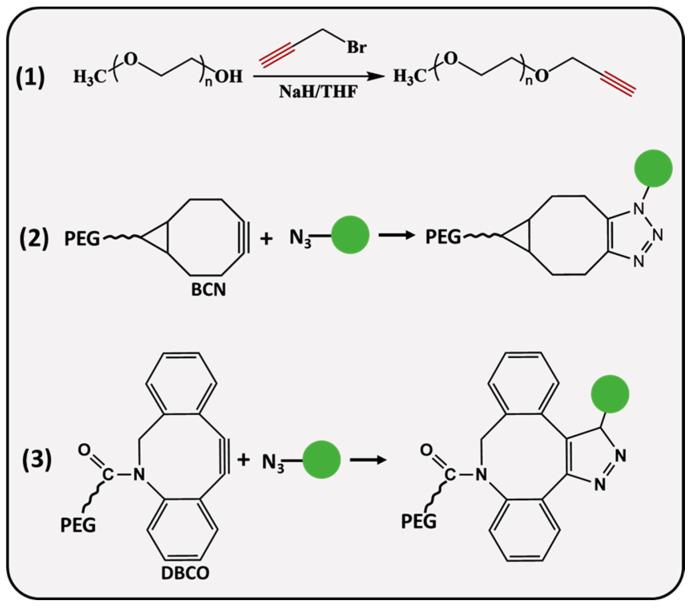
Synthesis and conjugation strategies of alkyne-functionalized PEG.

PEG molecules (Figure 2) can be modified with linear or strained alkynes (SCO), such as dibenzocyclooctyne amine (DBCO) and bicyclononyne (BCN). Linear propargyl PEG finds its application in copper-assisted azide-alkyne cycloaddition (AAC), while SCO-PEG is employed for copper-free AAC, also referred to as SPAAC. It is an excellent choice for in vivo applications reducing the potential cytotoxicity associated with the catalyst [34]. SPAAC is a biorthogonal click reaction that occurs rapidly under physiological conditions, avoiding interference with biological processes which makes it practical for cell imaging [35,36,37] and hydrogel preparation [38,39]. A recent study demonstrated a remarkable in situ application of SPAAC for a potential brain injury treatment. Fibrinogen, a key protein in blood clotting, was BCN-modified and intravenously delivered to the site of injury, where it was retained for up to 4 days, allowing further treatment with azide materials for improved therapeutic delivery [40]. He and coworkers designed a pH-sensitive, doxorubicin (DOX) attached DOX-PLA-PEG prodrug through the reaction of an azide-modified PEG-PLA and BCN-DOX, the in vitro drug release profile of self-assembled NPs showed 19.04% release after ≈6 days of incubation at a physiological pH, whereas 69% of DOX was released at a pH = 5.3 below 75 h [41]. During the synthesis of propargyl (PPG) derivatives, the hydroxyl group of PEG is deprotonated by a strong base (sodium hydride, potassium hydroxide) followed by a nucleophilic substitution reaction with PPG-bromide [42,43]. Herzberger et al. developed a novel procedure where glycidyl PPG ether was used as a monomer in anionic copolymerization with ethylene oxide [44]; the PPG groups were incorporated into the polymer chain without the alkyne group protection. Alternatively, Lu and colleagues employed an α-hydroxy-ω-carboxyl PEG; initially, the carboxyl terminus was converted to a PPG group through a reaction with PPG-bromide, whereas α hydroxyl ends were modified with amine, mercapto and hydrazide groups at the other end, resulting in PEG derivatives that are useful for site-specific bioconjugation applications [45].

### 2.3. Aldehyde- and Nitrophenylcarbonate-Functionalized PEG

**Figure 3:**
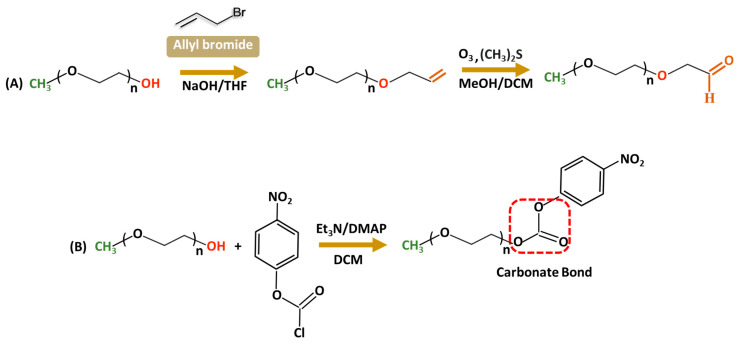
Synthetic routes for functionalized PEG derivatives from a terminal Alkene (**A**) or nitrophenyl chloroformate (**B**).

Aldehyde-modified PEG is largely used in conjugation with amine groups of biologically active molecules such as proteins [46] to increase stability and bioactivity. The reaction with a protein primary amine group forms a reversible Schiff base (C=N) that can undergo hydrolytic conversion into the original amine and carbonyl compound under acidic conditions. Stability issues are often resolved by reducing the Schiff base to an amine using a reducing agent such as NaBH_4_, forming stable C-N bonds. Only a few literature sources are available for PEG–aldehyde synthesis because of the challenges associated with hydroxyl groups’ low reactivity. The widely adopted method is the oxidation of the alcohol moiety [47,48]. In 2015, Mauri and colleagues reported the two-step synthesis of PEG–aldehydes; traditional oxidation methods often struggled with the low reactivity of PEG resulting in low yields with unwanted by-products. In contrast, in the new approach (Figure 3A), PEG was functionalized with terminal alkenes, followed by ozonolysis and subsequent reduction with dimethyl sulfide forming stable, reactive derivatives without changing the structural integrity of PEG [49]. In addition, *p*-nitrophenylcarbonate-PEG (NPC-PEG) represent a class of highly reactive electrophiles; they are synthesized through a reaction of nitrophenyl chloroformate (NPC) and PEG in common organic solvents—THF, DMSO, and DCM—in the presence of the base catalysts including triethylamine [50,51,52,53] or dimethylamino pyridine (Figure 3B) [54]. Generally, NPC is taken in a slightly excess molar ratio relative to PEG to ensure a high conversion of hydroxyl groups. Acylation is performed at low temperatures (0–5 °C) during the initial hours to increase the stability of NPC and minimize side reactions; nevertheless, the reaction is very sensitive to water, often accompanied by the formation of 4-nitrophenol as a major by-product of hydrolysis, which complicates the purification. The resulting NPC–PEG can be conjugated with various molecules such as dendrimers [55,56], or micelles forming stable urethane linkages. For protein conjugation, releasing p-nitrophenol can be easily detected using UV spectroscopy to evaluate the degree of PEGylation reaction.

**Table 1:** PEG derivatives and their application.

PEG Derivative	Primary Application	Reaction	Benefits
PEG-Amine	Protein, peptide conjugation	N-terminal, Lysine PEGylation	Increased stability and half-life [57,58,59]
PEG-Silane	Surface functionalization, drug delivery	Highly reactive with hydroxyl groups on surfaces	Stable surface functionalization, increased circulation time [60]
PEG-Aldehyde	Protein conjugation	Nucleophilic addition with hydroxyl or amine groups	Improved half-life [61,62]
PEG-Azide	Click chemistry	Click reaction with alkyne-functionalized molecules	High specificity, bio-orthogonality
PEG-Acrylate	Tissue engineering and hydrogel preparation	Michael addition, radical polymerization	Hydrogel scaffolds for 3D cell culture [63,64], wound dressing [65,66], tissue engineering [67]
PEG-Maleimide	Protein, drug, NP conjugation	Reacts with thiols (cysteine) in proteins, gold NP surface	Increased stability, half-life [68,69,70,71]
PEG-Nitrophenylcarbonate	Protein conjugation, crosslinking	Nucleophilic substitution reactions	Rapid and simple modification of nanocarriers for protein conjugation

## 3. PEGylation Strategies for Nanosystems

**Figure 4:**
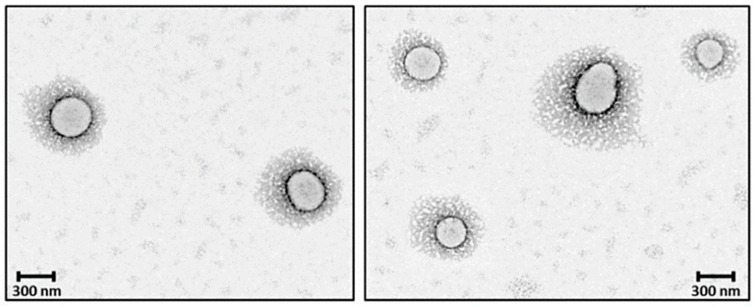
TEM micrographs core–shell PEGylated NPs. Reprinted with permission from Ref. [80].

PEGylation of nanosystems can be achieved through different methods, including covalent conjugation, physical adsorption and self-assembly. However, the stability of PEG-coated NPs can vary depending on whether PEG is attached to the NP surface. The PEG covalent binding to NPs is typically preferred to physisorption due to the strong nature of covalent bonds that can resist detachment under various conditions. For instance, Otsuka et al. demonstrated that gold NPs modified with α-lactosyl-ω-mercapto-PEG enhanced stabilization compared to those with physically adsorbed PEG [72]. Walkey et al. showed that the high density of PEG under covalent conjugation reduces protein adsorption and improves NP circulation in biological fluids by providing effective steric stability [73]. Physical adsorption of PEG is weaker and more susceptible to detachment; however, researchers have shown that the physisorption of cyclic PEG (c-PEG) onto silver NPs enhanced the dispersion stability against physiological conditions, light exposure and high temperatures by maintaining the antimicrobial activity of AgNPs [74]. Similarly, Wang et al. prepared c-PEG-stabilized gold NPs showing prolonged blood circulation and enhanced accumulation in tumor tissue [75]. Covalent PEGylation can be accomplished by direct conjugation of PEG molecules to the surface of the NPs, for example, PEG molecules with thiol groups can react with the metal NP surface like gold or silver with high affinity, forming stable Au-S [76,77] and Ag-S [78] bonds. Often PEGylation is performed through a non-direct approach, when PEG is first modified into amphiphilic forms as part of diblock copolymers like PEG-PLA and PEG-PCL or triblock copolymers [79] also known as pluronics, forming self-assembled structures on NPs. In our previous work, we reported the synthesis of PEG-grafted poly(ester amide) (PEG-PEA) using a thiol-Michael addition reaction. The NPs were prepared by a solvent-displacement method where the PEG-PEA was used as a surfactant, forming self-assembling structures (Figure 4), which were confirmed by transmission electron microscopy [80].

**Figure 5:**
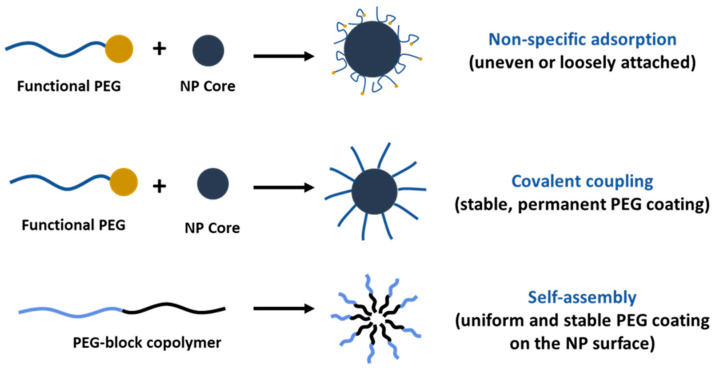
Comparison of PEGylation strategies for nanoparticle surface modification.

As a surfactant, PEG can stabilize emulsions, suspensions and other systems. However, only PEG cannot assemble on the NPs’ surface because of its hydrophilic nature, except when in combination with hydrophobic segments (Figure 5). PEG-containing copolymers can self-assemble into well-organized structures due to molecular interactions. In aqueous environments, hydrophobic segments of amphiphilic PEG tend to avoid water and interact with the core through Van der Waals forces or electrostatic interactions for the charged polymers [81]. The ratio of hydrophilic and hydrophobic blocks (Hp/Hb) and their molecular weight (Mw) can be adjusted to control the stability and biological properties of NPs. Ueya et al. showed that increasing the PEG ratio in a PEG-PLGA block copolymer decreased the stability of micellar NPs due to the improved hydrophilic nature, by increasing the Mw of PLGA, water diffusion into the core was hindered, resulting in enhanced stability [82]. The ratio of Hp/Hb blocks of PEG-PLA was shown to affect the cellular internalization of self-assembled NPs in HepG2 tumorcells. The results indicate that clathrin-mediated endocytosis was the predominant pathway for micelles with a 5:5 Hp/Hb ratio, whereas micelles with an Hp/Hb ratio closer to 2:8 showed the lowest cellular uptake, which was attributed to the formation of a less dense protein corona, and therefore better cellular uptake [83]. PEG content can significantly influence the drug release profile from NPs, higher concentrations usually result in faster and more extensive release due to the increased hydrophilicity of the NP surface as a result of enhanced water uptake that facilitates faster drug release [84,85,86]. Certain structures, like micelles, form self-assembly structures above a specific concentration known as the critical micelle concentration (CMC), which is the level of surfactant molecules in a solution above which they spontaneously form organized structures; high-CMC surfactants form micelles that are less stable and more susceptible to dissociation when diluted [87]. The PEG-block copolymer surfactants tend to have lower CMC values compared to conventional surfactants. Long hydrophobic blocks lower the CMC [88] as they enhance the tendency for self-assembly by reducing solubility; meanwhile, in turn, a long PEG block increases the CMC. However, a balanced ratio is important to decrease the CMC and maintain other physical properties which are necessary for drug delivery purposes.

## 4. Flory Radius, PEG Chain Length, and Density: Influence on PEG Conformation and Biological Interactions

**Figure 6:**
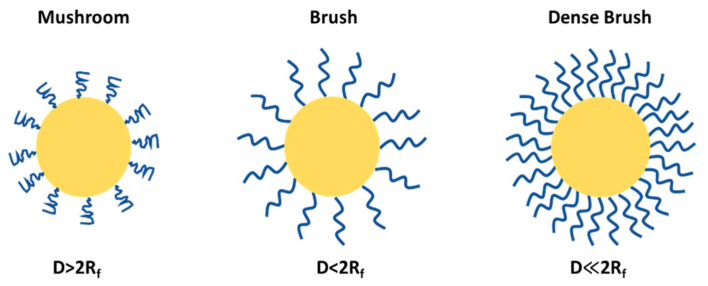
PEG conformational regime on NP surface.

PEGylation can lead to mushroom and brush structures on the NP surfaces depending on factors such as solvent, molecular weight and density of PEG chains (Figure 6). The conformational regime of PEG can be predicted from the Flory radius using the following relation: R_f_ = αN^3/5^, where N is the number of the monomeric units in the PEG polymer chain and α is the length of the monomeric unit (0.35 nm), and the exponent value (3/5) serves only for a good solvent and may vary depending on the solvent type and the Mw of the PEG. The grafting density or the average distance between adjacent PEG chains is calculated by the following formula D = (A/π)^1/2^, where A is an area occupied in NP occupied per PEG chain; using these two formulas one can predict the PEG conformation, and whether the PEG adopts a brush-like (D < 2R_f_) or mushroom-like (D > 2R_f_) structure. At a higher PEG density, in a brush configuration, the PEG chains are extended and occupy more space due to higher R_f_ relative to D and vice versa in a mushroom configuration; the chains are more compact and the effective size is smaller.

**Figure 7:**
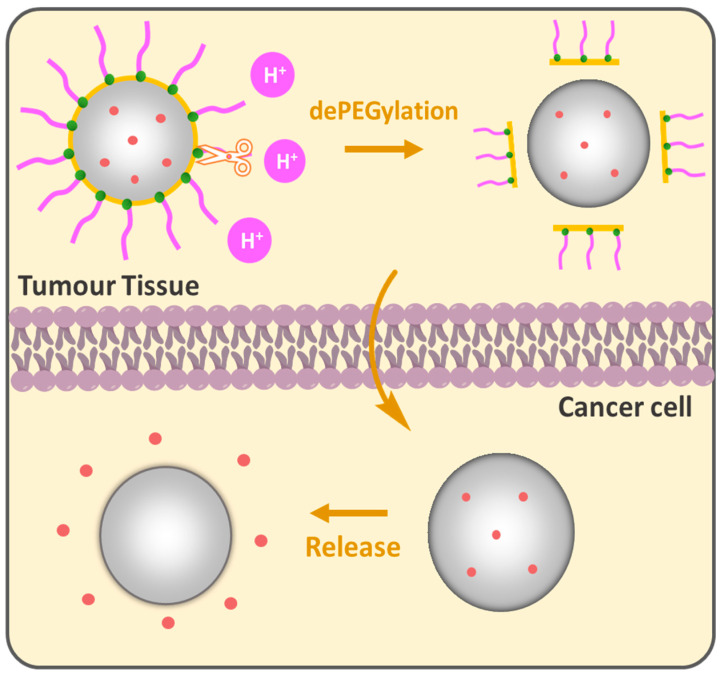
pH-responsive dePEGylation of nanoparticles for drug delivery.

The PEG cloud creates a “hydrophilic shield” that prevents opsonization-coating by plasma proteins that signal for phagocytosis by the reticuloendothelial system (RES), the bulky nature of PEG groups sterically hinders interactions providing NP stability in biological environments. In this regard, the grafting density of PEG is a key parameter for the colloidal stability of NPs [89,90]. High PEG surface density is generally preferred for biological applications that show biocompatibility and low protein adsorption [91] inhibiting phagocytic uptake [92]. However, as brush conformation avoids phagocytic uptake, it does not necessarily guarantee full protection from macrophage capture. It was shown that a minimal brush PEG coating could not evade THP-1 macrophage detection and sometimes dense brush conformation is required for an effective reduction in protein adsorption [93] and macrophage uptake [94]. Wang et al. showed the importance of PEG chain size in the comparative study while the terminal PEG density was constantly regulated by changing the PEG-PCL block copolymer molecular weight in PEG_n_-PCL_n_/PCL_3,5k_ core–shell NPs. Increasing the PEG length from 10.7 nm (3.4 kDa) to 13.8 nm (5 kDa) improved the tumor growth inhibition from 73.4% to 88%; however, a further increase in the PEG length to 24.5 nm reduced efficacy to 54%. PEG 5 kDa, particularly between PEG 3.4 kDa and PEG 8 kDa, showed enhanced “stealth” properties, longer circulation time and reduced macrophage interactions, which indicates that optimizing the PEG length is crucial for designing effective DDSs [95]. Gref et al. studied the impact of PEG Mw in PEG-PLA (45 kDa)/PLA_40k_ core–shell NPs on protein adsorption. A steep decrease was observed when increasing PEG Mw from 2000 to 5000 g/mole. PEG 5 kDa was found to be optimal for protein resistance and no significant benefits were observed when increasing PEG Mw above 5000 compared to bare PLA_40k_ NPs. The reduced protein adsorption for PEG content in PEG_5k_-PLA_20k_/PLA_40k_ NPs was between 2 and 5 weight %; however, no complete protection against plasma protein adsorption was achieved [96]. While long PEG chains improve stability and circulation, they can also inhibit the cellular uptake of the NPs [97,98,99], which brings us to the ”PEG dilemma”. The conventional DDSs are PEGylated to acquire the ”stealth” mode, providing a variety of benefits; nevertheless, many PEGylated formulations encounter limitations such as low cellular uptake and effective targeting to specific locations, especially when it comes to tumors. Compared to normal cells, cancer is distinguished by a unique microenvironment with lower pH, elevated temperature and the presence of specific enzymes. These properties can be purposefully leveraged to design PEGylated DDSs. The idea of dePEGylation has been developed as a responsive tool to counteract the abovementioned limitations of PEGylation. The principle lies in the following: NPs remain “stealthy” during circulation to protect the drug and dePEGylation occurs only after they reach the target tissue, releasing active components (Figure 7). pH-responsive dePEGylation is often performed through acid-sensitive bonds such as hydrazine [100], β-thiopropionate [101] and acetal/ketal [102]. In 2003, Shin et al. synthesized acid-labile DOPE-liposomes loaded with fluorescent calcein; the authors demonstrated that the release was influenced by PEG molecular weight. Higher PEG Mw provided slower calcein release rates compared to lower ones and the dePEGylation strategy improved overall release compared to its permanently PEGylated analogues [103].

Ideally, fully stealth NPs should avoid opsonization and macrophage uptake, leading to prolonged circulation and monophasic clearance of NPs; however, in practice, it is rare. Many DDSs, including PEGylated systems, show an initial sharp α-phase clearance, which occurs shortly after administration; therefore, these materials cannot indiscriminately be classified as stealth but rather pseudo-stealth because the complete evasion of the immune system remains challenging. A “true stealth effect” appears to be further complex, as it is influenced by multiple factors beyond just surface modification with PEG, physicochemical properties such as NP size, shape, surface charge and the dynamic formation of the protein corona play significant roles in immune recognition and clearance [104]. Interestingly, Li and coworkers developed enzymatically transformable NPs. It is known that matrix metalloproteinases (MMPs) are often associated with the formation of new blood vessels to supply the growing tumor. In a new strategy, MMPs were employed in tumor-associated tissues to deliver marimastat (metalloprotease inhibitor) and colchicine (anti-inflammatory) drugs. PEGylation significantly prolonged the blood circulation half-life and the nanotherapeutic remained stealthy in the bloodstream, with a half-life of approximately 7 h. MMP-2-governed DePEGylation accelerated the release of internal marimastat, with approximately 70% released within 12 h. Co-delivery treatment showed a reduction in pulmonary metastases, with over 80% of mice being metastasis-free; meanwhile, only colchicine-loaded NPs or co-delivery of non-transformable NPs (i.e., unable to dePEGylate) did not show significant suppression [105]. A novel light-triggered PEGylation/dePEGylation strategy was reported by Zhou and coworkers, which utilized near-infrared light to activate iRGD, a peptide that enhances drug accumulation and penetration into the tumor [106].

## 5. Quantification of PEG Surface Density on Nanoparticles

**Figure 8:**
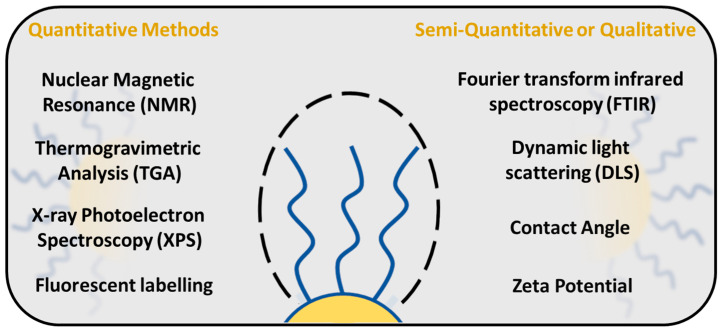
PEG surface density quantification methods.

There are several techniques available to study and evaluate PEG density around the NP surface (Figure 8). Quantification of PEG surface density is important for determining the PEG conformational regime, especially in the area of drug delivery, which, as shown before, directly influences the NP properties such as stability, solubility and interaction with the immune system. One of the methods to study the PEG surface density is a fluorescence-based assay, in which PEG molecules are labelled with a fluorescent dye, and the PEG surface density is measured by the fluorescence intensity of the dye [88,107]. NPs are typically incubated with fluorescent dyes, followed by a washing step to remove unbound labels. The signal is measured and the PEG surface density is calculated from the standard calibration curve.

X-ray photoelectron spectroscopy (XPS) examines the chemical and elemental states of a material surface, and it is commonly used to confirm the successful PEGylation of the NP surface. The existence and relative abundance of oxygen and carbon atoms indicate the presence of PEG molecules on the NP surface to analyze the PEG cloud around the NPs [25,28,29,32,76,108]. XPS is particularly useful for verifying the modification of NPs with PEG and its efficacy in reducing protein adsorption [109]. The surface density of PEG can be calculated by comparing the intensity of the PEG-related peaks to the total surface area of the NPs [110]. However, XPS is a surface-responsive technique that usually covers up to 10 nanometers of the sample; therefore, the experimental results correspond to the outer region of the material, and PEG coverage below this surface region cannot be detected.

Thermogravimetric analysis is a useful technique for evaluating the PEG density on a NP surface. The PEG number is quantified by analyzing the weight loss as a function of temperature, [76,108,111,112] based on the calibration of the pure PEG molecule which has been used for NP preparation. However, TGA provides information about the total PEG content, but does not directly reveal how PEG molecules are distributed on the surface of the NPs [113].

NMR spectroscopy provides quantitative information about the surface density of PEG on NPs. The average number of PEG molecules for each NP and chain per surface area can be calculated from ^1^H-NMR spectra by integration of the CH_2_ groups of the PEG peak typically observed at ~3.5 ppm, which can be compared to the internal [76] or the external standard signal [92]. The conformation of PEG chains can vary from mushroom to brush conformations that impact the mobility and dynamics of the polymer chains. Moreover, the measurement of the spin relaxation time promotes a better understanding of PEG chain conformation around the NPs, and with the increased mobility of the polymer chains, the relaxation time decreases [92].

## 6. Protein PEGylation

### 6.1. N-Terminus and Lysine PEGylation

PEG derivatives can be conjugated to various functional groups in biomacromolecules. For protein modification, the PEGylation of amino groups is preferred because of its abundance and versatility (i.e., the ability to react with different PEG-linkers). Site-specific N-terminal PEGylation and lysine (Lys) PEGylation are the two most important techniques. The N-terminus is modified through maleimide or NHS ester-activated PEG. Peng and colleagues showed the covalent attachment of PEG 5 kDa molecules to immunostimulatory peptide Thymosin alpha 1 (Tα1); peptides with PEG attached to the N-terminus exhibited a longer half-life and improved immune activity compared to cysteine thiol-modified analogues (Cys-Tα1), attributed to the degradation of Cys-Tα1 over time as a result of interactions between the peptide and serum proteins [68]. Yu et al. reported remarkable N-terminal PEGylation of recombinant human interleukin (rh-IL11), increasing the half-life approximately from 3 h for non-PEGylated rh-IL11 to 67 h for PEGylated-IL11 during subcutaneous injections in monkeys [61]. On the other hand, Lys-PEGylation has been proven to be effective against proteolytic degradation of protein fibronectin (FN), playing a major role in cell adhesion, growth, migration and differentiation. The authors studied the influence of PEG molecular weight on FN stability and biological activity. Cell properties (adhesion, cell spreading) deteriorated by increasing PEG Mw [114]. PEG conjugation to Lys side chains in the enzyme uricase is a popular technique for gout treatment, providing increased solubility and improved stability while reducing blood uric acid [115]. Similarly, Lys PEGylation is employed for the modification of interferon alfa-2a in combination with the antiviral medicine ribavirin for chronic hepatitis C treatment [116]. The method is also suitable for the functionalization of nitrophenylcarbonate-PEG-bound liposomes and micelles with different proteins for targeted drug delivery. The reaction is conducted at a slightly basic pH (7.5–8.5), and the residual by-products are removed by dialysis [117,118,119].

### 6.2. Cysteine (Thiol) PEGylation

Cysteine PEGylation has demonstrated effectiveness in modifying enzymes and monoclonal antibodies for improved tumor targeting [120,121]. The method is based on maleimide chemistry, where thiol groups bind to PEG-maleimide under neutral or slightly basic buffers, creating strong thioether bonds. The site-specific modification of G-CSF provided prolonged biological activity during neutropenia in a murine model. Interestingly, the high molecular weight PEG was associated with enhanced leukocyte proliferation [70]. Doherty and colleagues demonstrated that PEGylation notably enhanced the circulating half-life of G-CSF compared to its native form, and the half-life of PEGylated proteins was extended by increasing the molecular weight of PEG [71]. A similar finding was observed in a separate study [65]. Beyond G-CSF, thiol PEGylation has been employed to crosslink the enzyme L-asparaginase used in leukemia treatment. PEGylation increased the enzyme’s hydrodynamic radius, which reduces the probability of glomerular filtration. Additionally, intramolecular crosslinking with PEG stabilized the enzyme’s active region and improved the catalytic activity [122].

### 6.3. Carboxyl PEGylation

**Figure 9:**
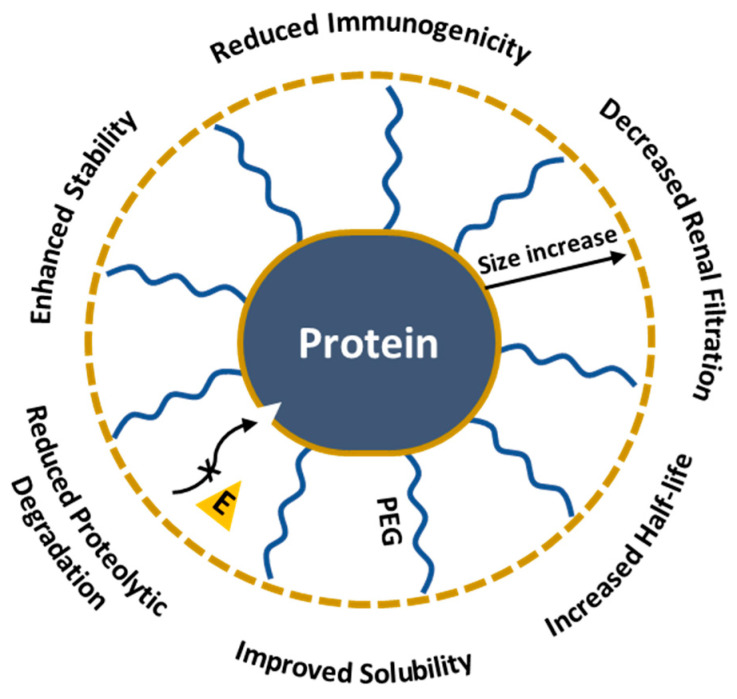
Impact of PEGylation on protein therapeutics.

Carboxyl PEGylated products show enhanced solubility and reduced immunogenicity [123]. PEGylation specifically targets aspartic and glutamic acid residues through 1-ethyl-3-(3-dimethylaminopropyl) carbodiimide (EDC) treatment. Initially, the COOH group in the protein is activated by EDC, converting the carboxyl group into an O-acylisourea reactive form that facilitates the reaction with the primary amine groups of PEG. The 1997 study shows the first carboxyl-specific PEGylation of BDNF neuropeptide, essential in neuron survival, growth and differentiation. PEGylation improved the pharmacokinetic properties of the peptide and decreased plasma and hepatic clearance; however, the authors reported increased renal clearance of BDNF-PEG_2000_ compared to non-PEGylated BDNF, which they attributed to the peptide’s enhanced cationic properties as a result of PEGylation. Despite the cationic nature, BDNF-PEG_5000_ showed a decrease in removal, underlying the importance of peptide size in drug development [124]. In conclusion, PEGylation is a powerful tool for creating new protein therapeutics due to its ability to enhance half-life, solubility and reduce the degradation and clearance of proteins (Figure 9). Nevertheless, PEG could interfere with the protein’s active site or overall structure and might alter the bioactivity [125,126,127,128]; therefore, selecting the appropriate modification method is important for maintaining the protein’s functionality.

## 7. PEG Immunogenicity

PEGylation enhances drug properties by increasing circulation time, improving stability and solubility; however, the repeated use of PEG has led to an unexpected consequence, the development of anti-PEG-antibodies. The immune system’s recognition of PEG appears to be a complex mechanism. When the body encounters PEGylated compounds, immune cells can generate antibodies specifically targeting PEG epitopes. These antibodies may be of different isotypes, predominantly IgM [129,130,131] and IgG [132] followed by a quick removal and immune responses. Studies have revealed the prevalence of APAs in the general population, with 25% of healthy blood donors possessing it [133]. In another study, APAs were detected in 44.3% of healthy donors, where both antibodies IgM (27.1%) and IgG (25.7%) were significantly more common in females as compared to males, which is in accordance with the separate study [134], likely due to cumulative exposure to PEG-containing products in daily life. The presence of these antibodies has been associated with the accelerated blood clearance (ABC) phenomenon, reduced therapeutic efficacy and potential hypersensitivity reactions in patients receiving PEGylated medications [8,135]. Several key factors influence the development of anti-PEG immunity and the ABC phenomenon such as previous exposure to PEG, administration routines, PEG characteristics, etc. Münter et al. compared the antibody generation against PEGylated mRNA-carrying NPs by different administration routes. Intramuscular injections in mice were found to generate overall low- and dose-independent levels of antibodies, while intravenous and subcutaneous injections generated substantial levels of IgG and IgM antibodies [136]. In separate research by Takata et al., all tested routes for mPEG_2000_-DSPE induced anti-PEG IgM production; however, the maximum production of anti-PEG IgM antibodies was observed after intravenous administration and anti-PEG IgM production was significantly reduced after splenectomy for all administration routes, indicating the spleen’s key role in antibody generation [137]. Opinions vary on how PEG density affects the ABC phenomena, for example, Li et al. reported that liposomes with a higher PEG grafting density (e.g., 9%) tend to induce an enhanced ABC phenomenon compared to those with a lower PEG density (e.g., 3%). Both high- and low-PEG formulations induced similar levels of anti-PEG IgM following the first dose. However, the higher PEG density results in stronger recognition and neutralization during subsequent doses, leading to faster clearance [138]. On the contrary, Ishida et al. showed that a PEG surface density of 5 mol% on mPEG_2000_-DSPE liposomes was found to induce the ABC phenomenon at the first dose, while the PEG density way below or higher this threshold reduced the ABC phenomenon. The authors have suggested that a high density of PEG reduced the activity of splenic B cells, resulting in less clearance of the subsequent dose and increasing PEG Mw from 2000 up to 5000 g/mol, and did not affect the ABC phenomenon [139]. In addition to PEG Mw, the immunogenicity of PEG is influenced by its terminal group [140,141]. The molecular basis of anti-PEG antibody formation involves complex immunological mechanisms. Research by Ishida et al. has shown that empty PEGylated liposomes induce an anti-PEG IgM response in rats and mice, even in T-cell-deficient mice, indicating that the response is T-cell-independent [142]. APA detection methodologies have evolved significantly, to name some of them, immunosorbent assay ELISA, flow cytometry and surface plasmon resonance. Ehlingeret et al. demonstrated high sensitivity in detecting clinically relevant anti-PEG antibody levels in a large analysis involving 200 healthy patients. The ELISA method detected 97.5% of pre-existing antibodies in human serum [143]. The existence of anti-PEG antibodies remains challenging in the vaccination process, cases of immediate hypersensitivity reactions including anaphylaxis were reported following the administration of PEG-containing COVID-19 vaccines, particularly those employing LNPs. In the study, 130 adults received either the BNT162b2 (Pfizer-BioNTech) or mRNA-1273 (Moderna) mRNA vaccines against SARS-CoV-2, ELISA analysis showed anti-PEG IgG was detectable in 71% of subjects prior to vaccination. However, APAs were boosted after two doses of vaccination: Pfizer-BioNTech showed a tiny 1.78-fold change for IgG and 2.64 for IgM, while the Moderna vaccine showed 13.1 for IgG and 68.5 for IgM and no increase was observed in unvaccinated controls [134]. In 2023, Kozma et al. studied the potential role of APAs in allergic reactions to PEG COVID-19 vaccines; the results showed elevated levels of IgG and IgM antibodies indicating that individuals with higher levels are more likely to experience allergic reactions upon receiving PEG-containing vaccines [144].

## 8. PEGylated Nanocarriers in Cancer Therapy

### 8.1. PEGylated Liposomes

PEGylated liposomes are lipid bilayers coated by PEG on the surface, which reduces aggregation and enhances the colloidal stability of the formulation. Due to the enhanced permeability and retention effect (EPR), they accumulate more effectively in tumor tissues than in healthy ones. Klibanov et al. were among the first who reported the positive effects of PEGylation on liposomes, which were capable of evading RES detection, resulting in prolonged circulation time in the bloodstream [145]. New strategies have been developed to increase the efficacy of liposomal DDSs, such as pH-sensitive systems, which contain acid-labile bonds. They remain stable under the physiological pH of healthy tissues and the blood but degrade in acidic conditions of the tumour microenvironment where encapsulated drugs are released [146,147,148,149]. DOXIL is the most widely used PEGylated liposomal formulation of the chemotherapeutic drug DOX, approved for the treatment of breast cancer, ovarian cancer and multiple myeloma (Table 2). Safra et al. reported that 500 mg/m^2^ of DOXIL significantly reduced the cardiomyopathy risk which is generally associated with the use of free DOX [150]. Several methods have been used for the fabrication of liposomal structures, such as the pre-insertion method, where PEGylated lipids are incorporated into the formulation before the liposome preparation. Since the PEGylated lipids are present throughout the formation process, the resulting nanocarriers have a consistent surface modification but a lower PEG surface density [151]. Meanwhile, in the post-insertion method, liposomes are first formed without PEGylation. Afterwards, PEG–lipid conjugates are added to the pre-formed liposomes, and the PEGylated lipids spontaneously integrate into the liposomal bilayer due to hydrophobic interactions. Repeated administration of several PEGylated liposomes has been reported to lead to the generation of APAs, resulting in faster clearance [152,153,154,155]. Additionally, liposomes are limited by their drug-loading ability, purification steps and high production costs.

### 8.2. PEGylated Lipid Micelles

**Figure 10:**
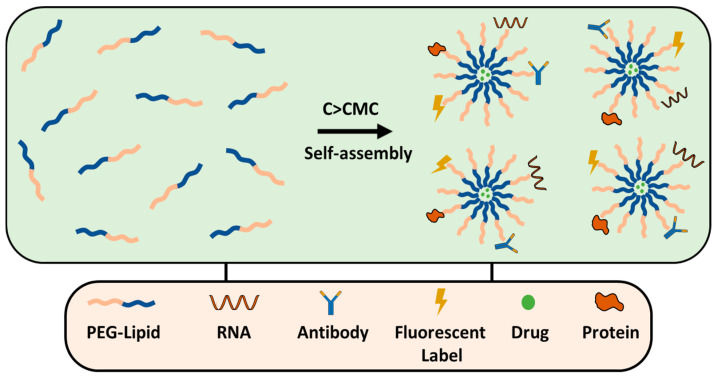
PEG-lipid micelle formation and conjugation strategies.

Lipid micelles are self-assembled structures formed by amphiphilic surfactants, such as PEG–DSPE, DOPE–PEG, PEG–cholesterol, etc. They consist of a hydrophilic outer PEG shell and a hydrophobic lipid core that can encapsulate poorly soluble drugs (Figure 10). PEGylated micelles, similar to liposomes, are effective in solid tumors; they typically range from 20 to 100 nm in size and are ideal for passive targeting via the EPR effect. On the other hand, micelles are used for active targeting, which is achieved by functionalizing with ligands (e.g., antibodies, peptides, or small molecules) that specifically bind to overexpressed receptors on the cancer cells. PEGylated micelles are prepared by thin-film hydration; generally, the drug and polymer are dissolved in an organic solvent, typically chloroform, or methanol, the organic solvents are removed by rotary evaporation and a produced thin film is hydrated forming micelles with the drug trapped inside. The method has been successfully used for the preparation of transferrin-targeted PEGylated liposomes for treating ovarian carcinoma in vivo models [156,157].

### 8.3. PEGylated Dendrimers

Dendrimers are highly branched, nano-sized three-dimensional polymeric structures with unique properties; they increase the solubility and the half-life of poorly soluble drugs [158,159,160]. Positively charged dendrimers are widely used in drug delivery, reacting with the negatively charged cell membrane; however, the charge on the surface also causes cytotoxic effects [161] and rapid clearance. The interaction of dendrimers with biological membranes can lead to membrane disruption by creating small holes [162]. PEGylation of cationic dendrimers is an effective way of overcoming cytotoxicity problems [163,164,165]. The PEGylation of the poly(amidoamine) dendrimer (PAMAM) was shown to improve the solubility and the anticancer activity of piperlongumine drug in human colon cancer with sustained drug release [166]. In another study, PEGylated DOX-loaded dendrimers showed high accumulation in C26 murine colon tumor cells (around 20 times) and reduced accumulation of DOX/dendrimers in the spleen compared to the free drug [167]. Besides reducing toxicity, PEGylation enables the further modification of dendrimers with different ligands for targeted delivery [168]. Dual-responsive PEGylated dendrimers have shown promise in glioblastoma treatment by improving the delivery of therapeutic agents across the blood–brain barrier (BBB), and studies in the C6 glioma cell line have demonstrated that ligand-modified PAMAM dendrimers conjugated with drugs like DOX and tamoxifen can increase their half-life, accumulate in brain tumors and reduce non-specific toxicity [169,170,171,172].

### 8.4. PEGylated Polymeric Nanoparticles

The amphiphilic nature of PLA-PEG, PLGA-PEG and the other copolymers governs their self-assembly in aqueous environments through different molecular interactions. Depending on the preparation technique and the hydrophilic-to-hydrophobic block ratio, these copolymers can form various nanostructures. Core–shell organizations (Figure 4) feature a hydrophobic inner polymer core, while the shell is made of hydrophilic PEG copolymers (i.e., PLGA(core)/PLGA-PEG(shell)). In contrast, micelles are amphiphilic PEG copolymers that spontaneously assemble without the distinct separate inner core seen in core–shell structures. Another interesting representative is the polymersome, which is a vesicular structure with a copolymer bilayer membrane. These structures are formed when the hydrophilic fraction of the copolymer is approximately 25–40% [173]. For drug delivery applications, therapeutic agents can either be encapsulated during the preparation process or covalently conjugated to the PEG-copolymer using stimuli-responsive linkers for controlled release. Because of the polymer’s high molecular weight NPs can encapsulate a high proportion of both hydrophobic and hydrophilic drugs, protecting them from degradation and providing sustained release. In addition, the NP’s PEG surface can be modified with various ligands, such as antibodies or proteins, that can specifically target receptors overexpressed on cancer cells. However, “naked” NPs are easily recognized by the immune system, when foreign bodies, including NPs, enter the bloodstream, the immune system quickly detects and removes them by phagocytosis [1]. PEGylation of the NP improves its stability, biocompatibility and circulation time [174]. PEGylation was shown to increase the water solubility and bioavailability of hydrophobic drugs [175]. The size of PEGylated polymeric NPs is one of the most important factors that determines the NPs’ behavior. NPs in the 10 nm to 200 nm size scale are optimal for efficient cellular uptake, tissue penetration and circulation in the bloodstream, as most of the NPs around 10 nm are filtered by the kidneys. As is known, the stability of non-modified NPs highly depends on a surface charge [176]. PEGylation of NPs appears to reduce their zeta potential [177,178,179]; however, the fact does not present a significant issue as PEG prevents NP aggregation through steric hindrance rather than the electrostatic stabilization mechanism. PEGylation affects interactions between NPs and cell membranes and their subsequent cellular internalization [180]. Generally speaking, amphiphilic NPs are internalized inside the cells through endocytosis, for particles with diameters up to 200 nm clathrin-mediated endocytosis has been a predominant pathway for cellular internalization [181].

#### 8.4.1. Methods for Preparation of PEGylated Polymeric Nanoparticles

##### Nanoprecipitation

Nanoprecipitation, also known as the solvent displacement method, is the most commonly used technique for the preparation of PEGylated polymer NPs (Table 3), which is very useful for encapsulating hydrophobic drugs; however, this process is less effective for hydrophilic drugs as precipitation is performed in the aqueous phase. NPs are prepared by adding an organic to the water phase; therefore, the organic solvent must be miscible with water. The polymer and the drug are dissolved in an organic solvent and slowly added to the aqueous phase under continuous stirring; the solvent can be removed by evaporation or dialysis. The particle size of PEGylated NPs can be controlled by modifying several key parameters such as polymer concentration, antisolvent volume-flow rate and stirring rate. The concentration of the polymer in the organic solvent impacts the size of the resulting NPs, generally speaking, the higher the polymer concentrations the larger particles are formed [182] and increasing the polymer concentration too much can lead to aggregation and finally precipitation. Normally, the nanoprecipitation is performed without the use of an external stabilizer as PEG itself can stabilize suspension, however, when NPs are freeze-dried, it is often associated with structural collapse and difficulty in reconstitution or changes in particles’ physiochemical properties, adding a small amount of stabilizer can prevent this issue [179,183,184,185,186]. The nanoprecipitation method is generally used for preparing self-assembled NPs, which consists of supersaturation, nucleation, growth and coagulation steps [187].

##### Single (O/W) and Double Emulsion (W/O/W) Solvent Evaporation Techniques

**Figure 11:**
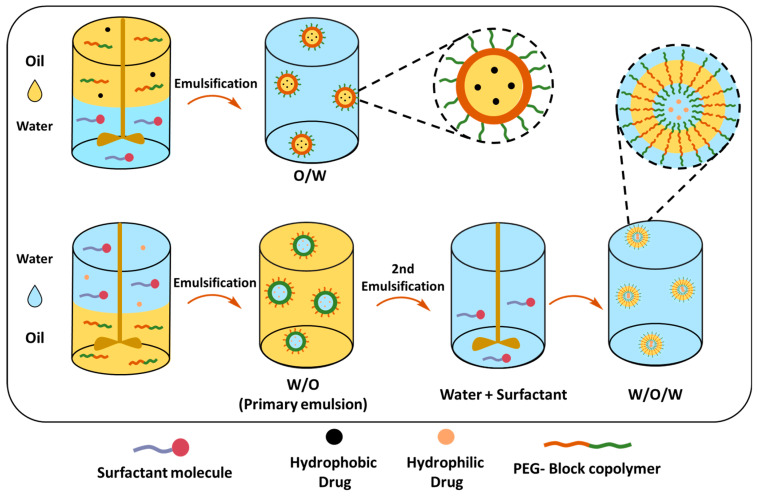
Single- and double-emulsion solvent evaporation methods for nanoparticle preparation.

The single-emulsion solvent evaporation method can mainly encapsulate hydrophobic drugs in PEGylated polymeric NPs (Table 4). The PEG-block-copolymer and hydrophobic drug are dissolved in a volatile organic solvent, which is added to the stabilizer-containing water under high-speed stirring or sonication, creating an oil-in-water emulsion (O/W) (Figure 11). The organic solvent can be removed by stirring at room temperature; however, the boiling point should be less than water to ensure a complete evaporation process. Resulting NPs can be collected by centrifugation, filtration or lyophilization. The size of the NPs similar to the nanoprecipitation method depends on several factors, such as the polymer concentration that affects the viscosity of the organic phase, as higher polymer concentrations typically result in larger NPs. The choice of organic solvent affects the final size of the PEGylated NPs, particularly, the evaporation rate of the solvent affects the rate at which the polymer precipitates to form NPs. DCM or chloroform are commonly used as they evaporate relatively quickly, promoting the rapid formation of NPs, slower evaporation rates, but on the other hand, allow the polymer to precipitate more slowly, leading to the formation of larger particles. Matsumoto et al. demonstrated that the release of the drug from NPs was affected by the PEG content in PLA-PEG-PLA and its molecular weight; copolymers with a higher PEG content (25.8%) showed faster drug release (95% in 24 h). In contrast, copolymers with a lower PEG content (5.2%) exhibited slower release (44% in 24 h); additionally, the lower Mw of copolymers and higher PEG Mw enhanced the initial and overall drug release [194]. In a different study, Xu et al. demonstrated the difference in TXL drug release profiles between micellar PLGA_45k_-PEG_5k_ NPs and the core–shell PLGA_45k_/PLGA_20k_-PEG_5k_ NPs; the latter showed sustained release, whereas it seems that the PEG content and matrix structure of the nanoparticle is detrimental for the drug release [195].

Whilst O/W is suitable for encapsulating hydrophobic drugs, the double-emulsion or water-in-oil-in-water (W/O/W) technique can be employed for encapsulating both hydrophilic and hydrophobic drugs (Table 5). In this technique, the drug is dissolved in water and emulsified in a polymer containing organic solvent, creating a water-in-oil (W/O) primary emulsion, that is further dispersed in the water phase, forming a W/O/W emulsion. The double-emulsion technique allows a more sustained and slow release of therapeutic reagents [177].

##### Emulsion Polymerization

**Figure 12:**
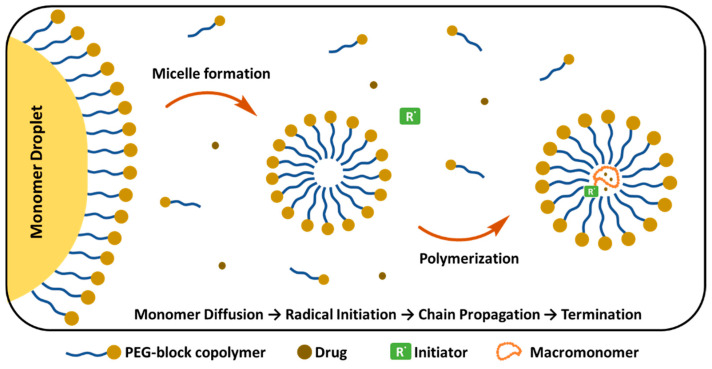
PEG-block copolymer micelle formation and drug encapsulation mechanism. (Figure created based on a template from BioRender.com (with modifications).

Emulsion polymerization (EP) takes place in the aqueous phase, where the hydrophobic monomers are initially presented as a “monomer droplet” (Figure 12). These droplets are stabilized by external surfactants or are self-stabilized by PEG copolymers that form micelles above the CMC. Various parameters, including the molar ratio of reagents and the molecular weight of the macromonomer, influence the final properties of PEGylated NPs [206]. In the initial stage, monomers diffuse inside the micelles and the polymer chain starts to grow. Simultaneously, PEG-containing components are incorporated into the growing polymer chains, creating a protective PEG corona around the NP. At the final step, chain growth is terminated and PEGylated polymer NPs are formed. Gurnani et al. synthesized biocompatible, core–shell PEGylated butyl acrylate NPs by RAFT emulsion polymerization technique, which showed long circulation time and did not require purification of the final product [207]. Similarly, EP was reported for the synthesis of surfactant-free PEGylated PCL NPs loaded with the anticancer TXL, in the absence of Cremphor, which is widely used for NP preparation, enhancing drug solubility. However, it also causes serious side effects including hypersensitivity and toxicity. NPs prepared by EP showed similar anti-tumoral activity to free TXL and the pharmacokinetic behaviour of encapsulated TXL showed no significant difference in drug recovery from urine and feces compared to free paclitaxel. In terms of safety and cancer targeting potential, these formulations offer advantages compared to free drugs [208]. Lupi et al. prepared PEGylated Rhodamine B labelled PCL NPs using an environmentally friendly two-step approach, combining ring-opening polymerization and water-based semi-batch EP, which is a surfactant-free process, and no organic solvents are required. PEGylation provided stealth properties, fast cellular uptake (within 1–2 h) and maintained steady concentration in tumors for 120 h [209]. Similarly, Ferrari et al. prepared PEGylated PLA NPs through surfactant-free and aided copolymerization of (hydroxyethyl)methacrylate (HEMA)-LA_4_ with HEMA-PEG macromonomers. After surfactant removal, NPs with short PEG chain length (<8.3 nm) precipitated; however, when PEG chain length was greater than 8.3, the PEGylated NPs remained stable in PBS solution, and no aggregation was observed. The surfactant addition reduced the final NP size to 30 nm. In the absence of surfactant, the diameter of the NPs tended to decrease, indicating that longer PEG chains give rise to smaller particles [210].

## 9. The Future Directions of PEGylation

We have entered the fifth decade of PEGylation research, and since the initial FDA approval, 41 PEGylated products have been listed on the market within 5 NP formulations, 2 small molecules and 34 biopharmaceuticals, such as hormones, enzymes, etc., and many of the PEGylated therapeutics are ongoing clinical trials. The future of PEGylation technology can be seen in the development of “smart” PEG systems that respond to environmental stimuli, such as enzymatic-, pH- and temperature-sensitive structures. Advances in bio-orthogonal chemistry, including click chemistry (SPAAC), further expand opportunities for PEGylated therapeutics. The development of branched, Y-shaped, U-shaped and comb-shaped PEG architectures refines the technology, which can provide similar or superior stability to linear PEG analogues [211]. The immunological impact of APA generation has pushed researchers into searching for alternative polymers with comparable pharmacokinetic benefits but reduced immunogenicity profiles, polymers such as polysialic acid, poly(N-(2-hydroxypropyl)methacrylamide), poly(2-oxazoline)s and poly(glutamic acid) derivatives show promise as potential PEG alternatives, as well as zwitterionic polymers, such as poly(carboxybetaine) and poly(sulfobetaine). These materials create a stronger hydration layer than PEG, potentially offering superior protein shielding while minimizing immunogenicity [212]. Studies have shown that with repeated administration of high-molecular-weight PEGylated products, PEG can accumulate in various organs, and vacuolation of cells has been observed in animal studies, particularly with higher molecular weight PEG [213]; this non-biodegradability of PEG also raises questions about its long-term use and the development of biodegradable alternatives or strategies to minimize accumulation will be important for the future of PEGylation technology.

## 10. Conclusions

In this review, we discussed the evolution of PEG from a simple aliphatic polymer to a sophisticated drug delivery system and demonstrated its benefits along with disadvantages. The molecular weight, architecture and density of PEG chains significantly impact the behaviour of PEGylated systems, and proper optimization of these parameters is required to enhance drug pharmacokinetics. Despite such benefits, anti-PEG immunogenicity and accelerated clearance of PEGylated drugs still remain challenging issues. Therefore, future research efforts should focus on novel conjugation chemistry, alternative polymer designs and improved formulation strategies that may help to overcome limitations while maintaining the beneficial part of PEGylation.

## Figures and Tables

**Table 2:** List of FDA-approved PEGylated liposomal formulations.

Brand Name	Active Ingredient	Cancer Type	Mechanism of Action
Onivyde	Irinotecan hydrochloride trihydrate	Pancreatic cancer, small-cell lung cancer, colon cancer	Topoisomerase I inhibitor
Doxil	Doxorubicin hydrochloride	Ovarian cancer, Kaposi’s sarcoma, multiple myeloma, breast cancer	Topoisomerase II inhibitor

**Table 3:** Characteristics of PEGylated NPs prepared by nanoprecipitation method.

Polymer	Encapsulated Drug	Organic Solvent	Antisolvent	EE or DL (%)	Stabilizer	Particle Size (nm)	Activity	Refs.
PLGA-PEG	Vinpocetine	Acetone	Water	60–90 ^●^	PVA	32–289	Cerebrovascular disorder	[178]
PLGA-PEG	Docetaxel	Acetonitrile	Water	-	Without stabilizer	153.3	Antineoplastic agent	[188]
PLGA-PEG	Ciprofloxacin	DMSO	Water	3–28 *	Without stabilizer	174–205	Antibiotic	[189]
PLA-PEG	pEGFP	Acetone	Water	-	CTAB/Tween 80	128.9	Plasmid vector	[190]
HA–PEG–PCL	DOXORUBICIN	Acetone	Water	95.56 ^●^	Pluronic F-68	95	Antineoplastic agent	[191]
Poly(isobutylcyanoacrylate/PCL-PEG	Busulfan	Acetone	Water	17.0 ^●^	Without stabilizer	152	Antineoplastic agent	[179]
PLGA-PEG	Platinum (IV)	Acetonitrile	Water	18.4 *	Without stabilizer	172	Antineoplastic agent	[192]
PLGA-PEG	Dexibuprofen	Acetone	Water	85–100 ^●^	PVA	201–226	Anti-inflammatory drug	[193]

* Drug loading (DL), ^●^ Encapsulation efficiency (EE).

**Table 4:** Characteristics of PEGylated NPs prepared by a single emulsion-solvent evaporation method.

Polymer	Encapsulated Drug	Organic Solvent	Emulsifier	Original Particle Size (nm)	Encapsulation Efficiency (%)	Refs.
PLGA-PEG	Cyclosporine	DCM	PVA	212	91.90	[196]
PLA-PEG-PLA	Progesterone	DCM	PVA	193–335	65–71	[194]
PLGA-PEG	Curcumin	Ethyl Acetate /DCM	PVA	152.37	73.22	[175]
PLGA-PEG	SN-38	DCM	PVA	249.2	81.85	[197]
PLA-PEG	Rhodamine B	DCM	PVA	169–201	31–68	[198]
PLGA–PEG	Curcumin	DCM	PVA	100–200	52.2	[199]

**Table 5:** Characteristics of PEGylated NPs prepared by double-emulsion solvent evaporation method.

Polymer	Encapsulated Drug(s)	Organic Solvent	Emulsifier	Particle Size (nm)	Encapsulation Efficiency (%)	Refs.
PLA-PEG	Tetanus toxoid	Ethyl Acetate	Sodium cholate	196	33.4	[200]
PLGA-PEG	Memantine	Ethyl Acetate	PVA	193–224	77–80	[201]
PLA-PEG-PLA	Methotrexate	Chloroform	PVA	100–173	23–48	[202]
PLA-PEG	Tetanus toxoid	Ethyl Acetate	Gelatin	136.8	35.3	[203]
PLGA-PEG	Bovine serum albumin	DCM	PVA	198.1	48.6	[177]
PLGA-PEG	Sorafenib + Doxorubicin	DCM/Acetone	PVA	177.2	8869	[204]
PLGA-PEG	Paclitaxel + Doxorubicin	DCM	PVA	243.63	70.1357.5	[205]
